# Supplementary material for: The transition to synchronization of networked systems
Source: Nat Commun. 2024 Jun 10;15:4955. doi: 10.1038/s41467-024-48203-6 (PMC11165003; doi:10.1038/s41467-024-48203-6)
Supplement: Supplementary file 1 — Supplementary Information [file 41467_2024_48203_MOESM1_ESM.pdf]

Supplementary Information
The transition to synchronization of networked systems

Atiyeh Bayani[1], Fahimeh Nazarimehr[1], Sajad Jafari[1,2], Kirill Kovalenko[3], Gonzalo Contreras-Aso[4], Karin Alfaro-Bittner[4], Ruben J. Sánchez-García[5,6,7], and Stefano Boccaletti[8,9,10]

[1]Department of Biomedical Engineering, Amirkabir University of Technology (Tehran polytechnic), Iran
[2]Health Technology Research Institute, Amirkabir University of Technology (Tehran polytechnic), Iran
[3]Scuola Superiore Meridionale, School for Advanced Studies, Naples, Italy
[4]Universidad Rey Juan Carlos, Calle Tulipán s/n, 28933 Mośtoles, Madrid, Spain
[5]Mathematical Sciences, University of Southampton, Southampton SO17 1BJ, UK
[6]Institute for Life Sciences, University of Southampton, Southampton, SO17 1BJ, UK
[7]The Alan Turing Institute, London, NW1 2DB, UK
[8]CNR - Institute of Complex Systems, Via Madonna del Piano 10, I-50019 Sesto Fiorentino, Italy
[9]Moscow Institute of Physics and Technology, Dolgoprudny, Moscow Region, 141701, Russian Federation
[10]Sino-Europe Complexity Science Center, School of Mathematics, North University of China, Shanxi, Taiyuan 030051, China

In what follows we consider a connected weighted undirected graph G with N nodes and uniquely identified by its adjacency matrix A and its Laplacian matrix $\mathcal{L}$. Furthermore, we will call $\lambda_1 = 0 < \lambda_2 \leq \lambda_3 \ldots \leq \lambda_N$ the (ordered in size) real and nonnegative eigenvalues of $\mathcal{L}$, and $\mathbf{v}_1 = \frac{1}{\sqrt{N}}(1, 1, 1, \ldots, 1)^T, \mathbf{v}_2, \mathbf{v}_3, \ldots, \mathbf{v}_N$ corresponding orthonormal eigenvectors. Finally, V will be the matrix having as columns the eigenvectors $\mathbf{v}_1, \ldots, \mathbf{v}_N$.

1 The clusters emerging in the transition to synchronization and the structural properties of the network

This is the main section of our Supplementary Information, where we give the mathematical proofs of the results stated in the main text and study the structural properties of the clusters observed during the transition to synchronization.

1.1 Spectral blocks and the proof of the main Theorem

Definition 1.1. *A subset $\mathcal{S} \subseteq \{\mathbf{v}_2, \ldots, \mathbf{v}_N\}$ consisting of $k-1$ Laplacian eigenvectors is called a spectral block localized at nodes $\{i_1, \ldots, i_k\}$ if*

- *each eigenvector from this set has all entries except $i_1, i_2, \ldots, i_k$ equal to 0;*
- *any eigenvector $\mathbf{v}_i$ not belonging to this set has the entries $i_1, i_2, \ldots, i_k$ all equal, i.e. $v_{i_1} = v_{i_2} = \ldots = v_{i_k}$.*

Note that, since all eigenvectors $\mathbf{v}_2, \ldots, \mathbf{v}_N$ are orthogonal to $\mathbf{v}_1$, the sum of all entries of the eigenvectors $\mathbf{v}_2, \ldots, \mathbf{v}_N$ has to be equal to 0.

Theorem 1.2. *Let G be a connected network with N vertices and Laplacian $\mathcal{L}$ and let $\{i_1, \ldots, i_k\} \subseteq \{1, \ldots, N\}$. Then the following two statements are equivalent:*

1. *All k nodes belonging to a cluster defined by the indices $\{i_1, \ldots, i_k\}$ have the same connections with the same weights with all other nodes not belonging to the cluster, i.e. for any $p, q \in \{i_1, \ldots, i_k\}$ and $j \notin \{i_1, \ldots, i_k\}$ one has $\mathcal{L}_{pj} = \mathcal{L}_{qj}$.*
2. *There is a spectral block $\mathcal{S}$ made of $k-1$ Laplacian eigenvectors localized at the nodes $\{i_1, \ldots, i_k\}$.*

In this case, starting from a given n, the (p,q) entries of the matrices S_n defined in the main text will be equal to 2, for all $p,q \in \{i_1,\dots,i_k\}$, $p \neq q$. Moreover, the eigenvalues corresponding to this spectral block will only depend on the subgraph induced by the nodes $\{i_1,\dots,i_k\}$ and the total added degree from all other nodes of the network.

Proof. Without loss of generality, we can assume $\{i_1,\dots,i_k\} = \{1,\dots,k\}$.

$\boxed{1 \Longrightarrow 2}$ Consider the $(k-1)$-dimensional subspace U formed by the vectors $\mathbf{u} = (u_1,u_2,\dots,u_N)^T \in \mathbb{R}^N$ for which $u_j = 0$ for all $j > k$ and the sum of all entries is equal to 0, that is,

$$U = \left\{ (u_1,u_2,\dots,u_N)^T \in \mathbb{R}^N \mid u_j = 0 \text{ for all } j > k \text{ and } \sum_{j=1}^{N} u_j = 0 \right\}. \tag{1}$$

This is indeed a $(k-1)$-dimensional subspace with (orthogonal) basis $\{\mathbf{e}_2,\dots,\mathbf{e}_k\}$, where $\mathbf{e}_i$ is a vector with non-zero entries 1 at position 1 and -1 at position i. We want to show that U is an invariant subspace of $\mathcal{L}$, that is, $\mathcal{L}\mathbf{u} \in U$ for all $\mathbf{u} \in U$.

Let $\mathbf{u} = (u_1,u_2,\dots,u_N)^T \in U$ and $j > k$. Then, the j-th entry of $\mathcal{L}\mathbf{u}$ is equal to

$$\sum_{i=1}^{N} \mathcal{L}_{ji} u_i = \sum_{i=1}^{k} \mathcal{L}_{ji} u_i. \tag{2}$$

By hypothesis, we have that $\mathcal{L}_{ji} = \mathcal{L}_{ij} = a$ for all $i \in \{1,\dots,k\}$, and hence

$$\sum_{i=1}^{k} \mathcal{L}_{ji} u_i = a \sum_{i=1}^{k} u_i = a \cdot 0 = 0. \tag{3}$$

It means that every entry of $\mathcal{L}\mathbf{u}$ after the k-th entry is 0. The second part (the sum of all the entries of the vector $\mathcal{L}\mathbf{u}$ is zero) holds for any vector: the column (or row) sum of $\mathcal{L}$ is zero, that is, $\mathbf{1}_N\mathcal{L} = \mathbf{0}_N$, which implies $\mathbf{1}_N\mathcal{L}\mathbf{u} = \mathbf{0}_N$ for any vector $\mathbf{u}$, where $\mathbf{1}_N$ (respectively $\mathbf{0}_N$) is a N-dimensional vector made of all entries equal to 1 (respectively 0).

Consider now L', the $k \times k$ principal submatrix of $\mathcal{L}$ given by the first k rows and columns. If $\mathbf{u} \in U$, then $\mathbf{u} = (\mathbf{u}'|\mathbf{0}_{N-k})^T$ and we can write, in matrix block form,

$$\mathcal{L}\mathbf{u} = \left(\begin{array}{c|c} L' & A \\ \hline B & C \end{array}\right) \left(\begin{array}{c} \mathbf{u}' \\ \hline \mathbf{0} \end{array}\right) = \left(\begin{array}{c} L'\mathbf{u}' \\ \hline B\mathbf{u}' \end{array}\right) = \left(\begin{array}{c} L'\mathbf{u}' \\ \hline \mathbf{0} \end{array}\right) \in U, \tag{4}$$

where $B\mathbf{u}' = 0$ since $\mathcal{L}\mathbf{u} \in U$. In particular, $\mathcal{L}\mathbf{u} = \lambda\mathbf{u}$ if and only if $L'\mathbf{u}' = \lambda\mathbf{u}'$.

Consider now the graph G' induced by the vertices $1,\dots,k$. Its Laplacian $L(G')$ equals L' except for the fact that it has diagonal elements d_i^{int} instead of $d_i = d_i^{\text{int}} + d_i^{\text{ext}}$. By hypothesis, $d_i^{\text{ext}} = d$ for all $i \leq k$. All in all, $L(G') = L' - d\,\mathbb{I}_k$ and, in particular, $(\lambda,\mathbf{v})$ is an eigenpair of $L(G')$ if and only if $(\lambda + d, \mathbf{v})$ is an eigenpair of L'.

Let $\mathbf{u}'_1,\dots,\mathbf{u}'_k$ be an orthonormal basis of the Laplacian $L(G')$ with $\mathbf{u}'_1$ a constant vector. In particular, the sum of the entries of each $\mathbf{u}'_2,\dots,\mathbf{u}'_k$ must be zero. If we define $\mathbf{u}_i = (\mathbf{u}'_i|\mathbf{0}_{N-k})^T$ for all $i = 2,\dots,k$, then such vectors are in the subspace U and, following the discussion above, $\mathbf{u}_2,\dots,\mathbf{u}_k$ must be eigenvectors of $\mathcal{L}$. Moreover, the eigenvalues corresponding to $\mathbf{u}_2,\dots,\mathbf{u}_k$ depend only on the structure of the induced graph G' and the external degree d.

Next, we show that every vector $\mathbf{v} \in \mathbb{R}^N$ orthogonal to $\mathbf{u}_2,\dots,\mathbf{u}_k$ must be constant on its first k entries. Indeed, since $\mathbf{u}'_2,\dots,\mathbf{u}'_k$ are linearly independent and U has dimension $k-1$, these vectors are also generate U. In particular, $\mathbf{v}$ must be orthogonal to all vectors in U. Recall (see above) that U has a basis $\{\mathbf{e}_2,\dots,\mathbf{e}_k\}$, and note that $\mathbf{v}\cdot\mathbf{e}_j = v_1 - v_j = 0$ implies $v_1 = v_j$ for all $k = 2,\dots,k$.

Therefore, if we complete the eigenvectors $\mathbf{v}_1,\mathbf{u}_2,\dots,\mathbf{u}_k$, where $\mathbf{v}_1$ is a constant unit vector, to an orthonormal eigenbasis $\{\mathbf{v}_1,\mathbf{u}_2,\dots,\mathbf{u}_k,\mathbf{v}_{k+1},\dots,\mathbf{v}_N\}$, the subset $\mathcal{S} = \{\mathbf{u}_2,\dots,\mathbf{u}_k\}$ necessarily forms a spectral block (Definition 1.1).

$\boxed{2 \Longrightarrow 1}$ Without loss of generality, we assume that the last $k-1$ eigenvectors form the spectral block localized at nodes $\{1,\dots k\}$. Then, the matrix V having the eigenvectors as columns can be written in block form as

$$V = \left(\begin{array}{c|c} C & V' \\ \hline V'' & \mathbf{0}_{N-k,k-1} \end{array}\right), \tag{5}$$

where V' and V'' are $k\times(k-1)$ and $(N-k)\times(N-k+1)$ matrices respectively, C is a $k\times(N-k+1)$ column-constant matrix ($C_{pj} = C_{qj}$ for all p,q,j), and $\mathbf{0}_{k-1,N-k}$ is a zero $(N-k)\times(k-1)$ matrix.

If Σ is the diagonal matrix of the eigenvalues $\lambda_1, \lambda_2, \ldots, \lambda_N$, we can write the Laplacian as

$$\mathcal{L} = V\Sigma V^T = \left(\begin{array}{c|c} C & V' \\ \hline V'' & \mathbf{0} \end{array}\right) \left(\begin{array}{c|c} D_1 & \mathbf{0} \\ \hline \mathbf{0} & D_2 \end{array}\right) \left(\begin{array}{c|c} C^T & (V'')^T \\ \hline (V')^T & \mathbf{0} \end{array}\right), \tag{6}$$

where D_1 (respectively D_2) is the diagonal matrix of the eigenvalues $\lambda_1, \ldots, \lambda_{N-k+1}$ (respectively $\lambda_{N-k+1}, \ldots, \lambda_N$), and $\mathbf{0}$ represents a zero matrix of the appropriate size. This implies that the connections between the nodes $\{1, \ldots, k\}$ and the nodes $\{k+1, \ldots, N\}$ are described by the top right submatrix

$$\mathcal{L} = \mathcal{V}\pm\mathcal{V}^{\mathcal{T}} = \left(\begin{array}{c|c} * & CD_1(V'')^T \\ \hline * & * \end{array}\right). \tag{7}$$

Finally, note that if C is a column-constant matrix, so is CM for any matrix M where the product exists. Or, explicitly, if $1 \leq p, q \leq k$ and $j > k$, then

$$\mathcal{L}_{pj} = \sum_{i=1}^{N-k+1} C_{pi}\Sigma_{ii}V''_{ji} + 0 = \mathcal{L}_{qj}. \tag{8}$$

For the final part of the statement, let us remind the (i, j) entry of S_n is equal to

$$\sum_{k=n}^{N} (v_{kj} - v_{ki})^2.$$

Let i, j be any two different nodes at which the spectral block $\mathcal{S}$ is localized. Then, if $\mathbf{v}_k$ does not belong to $\mathcal{S}$ the term $(v_{kj} - v_{ki})^2$ is equal to 0. Hence, the (i, j) entry of S_n changes only when $\mathbf{v}_n$ belongs to $\mathcal{S}$. So, if λ_n is greater than the maximum eigenvalue of $\mathcal{S}$ then the (i, j) entry of S_n is 0. On the other hand, if λ_n is less than the minimal eigenvalue of $\mathcal{S}$ the (i, j) entry of S_n is 2, as was to be shown. □

Actually, to be mathematically correct, the formulation of the second statement of the theorem should be *There is an eigenbasis $\mathbf{v}_1, \ldots, \mathbf{v}_N$ and a spectral block $\mathcal{S}$ localized at nodes $i_1, \ldots, i_k$*, since it could happen that eigenvectors not from U could have the same eigenvalues as eigenvectors in $\mathcal{S}$, so they could be not orthogonal to U. However, such a possibility does not affect the synchronization scenario, so we decided to omit to mention explicitly this extra case for the sake of simplicity.

One important example of equitable cells are symmetry orbits, that is, orbits under the action of the automorphism group of the graph. Note that, in real-world networks, most orbits are either complete or empty subgraphs with all permutations of the vertices realized as network symmetries [1, 4]. This particular case can be characterized as follows.

Theorem 1.3. *Let G be a connected network with N vertices and Laplacian $\mathcal{L}$ and let $\{i_1, \ldots, i_k\} \subseteq \{1, \ldots, N\}$. Then the following three statements are equivalent:*

1. *For any pair of vertices indexed in the set $\{i_1, \ldots, i_k\}$ a permutation of this pair preserves the Laplacian of the network.*

2. *The graph induced by $\{i_1, \ldots, i_k\}$ is either complete or empty, and for any $p, q \in \{i_1, \ldots, i_k\}$ and $j \notin \{i_1, \ldots, i_k\}$ one has $\mathcal{L}_{pj} = \mathcal{L}_{qj}$.*

3. *There is a spectral block $\mathcal{S}$ localized at nodes $i_1, \ldots, i_k$ and all $k-1$ eigenvectors of $\mathcal{S}$ have the same, degenerate, eigenvalue.*

In the proof of this theorem we will use the following proposition:

Proposition 1.4. *Let σ be a permutation of nodes $\{1, \ldots, N\}$ with permutation matrix P. Then the following two statements are equivalent*

1. *Permutation σ preserves $\mathcal{L}$, i.e. $P^{-1}\mathcal{L}P = \mathcal{L}$.*

2. *For any eigenvector $\mathbf{v}$ with eigenvalue λ, the vector $P\mathbf{v}$ is also eigenvector of $\mathcal{L}$ with the same eigenvalue λ.*

Proof of Proposition 1.4. $\boxed{1 \Longrightarrow 2}$ Let us consider any eigenvector $\mathbf{v}$ of $\mathcal{L}$ with an eigenvalue λ. Recall that a permutation matrix is invertible (in fact, orthogonal). Then

$$\mathcal{L}P\mathbf{v} = PP^{-1}\mathcal{L}P\mathbf{v} = P\mathcal{L}\mathbf{v} = P\lambda\mathbf{v} = \lambda P\mathbf{v}.$$

$\boxed{2 \Longrightarrow 1}$ Let $\lambda_1, \ldots, \lambda_N$ be the eigenvalues of $\mathcal{L}$ with corresponding orthonormal eigenvectors $\mathbf{v}_1, \ldots, \mathbf{v}_N$. Consider the associated spectral decomposition of $\mathcal{L}$, that is,

$$\mathcal{L} = V\Sigma V^{-1},$$

where Σ is a diagonal matrix of the eigenvalues and V the matrix with columns the corresponding eigenvectors. By hypothesis, $\{P\mathbf{v}_1, P\mathbf{v}_2, \ldots, P\mathbf{v}_N\}$ are also eigenvectors with the same eigenvalues $\lambda_1, \lambda_2, \ldots, \lambda_N$, and they are also orthonormal, since P is a permutation, hence orthogonal, matrix. As the matrix having $P\mathbf{v}_1, P\mathbf{v}_2, \ldots, P\mathbf{v}_N$ as columns is precisely PV, we can write

$$\mathcal{L} = (PV)\Sigma(PV)^{-1} = P(V\Sigma V^{-1})P^{-1} = P\mathcal{L}P^{-1},$$

and we are done. □

Proof of Theorem 1.3. Without loss of generality, we can assume $\{i_1, \ldots, i_k\} = \{1, \ldots, k\}$.

$\boxed{1 \Longleftrightarrow 2}$ One can permute vertices i, j if and only if for any vertex $p \in \{1, \ldots, N\}$ one has $\mathcal{L}_{ip} = \mathcal{L}_{jp}$. Hence, one can permute any $i, j \in \{1, \ldots, k\}$ if and only if for any $i, j \in \{1, \ldots, k\}$ and any $p \in \{1, N\}$ one has $\mathcal{L}_{ip} = \mathcal{L}_{jp}$. So, one just needs to show that vertices $\{1, \ldots, k\}$ form a clique. And indeed, for any $i_1, j_1, i_2, j_2 \in \{1, \ldots, k\}$ one has $\mathcal{L}_{i_1 j_1} = \mathcal{L}_{i_2 j_1} = \mathcal{L}_{i_2 j_2}$.

$\boxed{2 \Longrightarrow 3}$ Theorem 1.2 guarantees that one has a spectral block localized at nodes $1, \ldots, k$, and it remains to show that all the corresponding eigenvalues are the same. Define the subspace U as in the proof of Theorem 1.2. This subspace is generated by the vectors $\{\mathbf{e}_i\}_{i=2}^{k}$ such that for $\mathbf{e}_i$ entries number 1 and i are equal to 1 and -1 correspondingly, and all other entries are equal to 0. Let $\mathbf{u} = (u_1 \ldots, u_n) \in U$ be an eigenvector of $\mathcal{L}$ with an eigenvalue λ. Since $\mathbf{u}$ cannot be constant (the sum of all its entries must be equal to 0), one can choose $j, l \in \{1, \ldots, k\}$ such that $a = u_j \neq u_l = b$. Since $1 \Longleftrightarrow 2$, we know that the permutation $\sigma = (jl)$ of nodes j and l with permutation matrix P preserves the Laplacian. Using the proof of Proposition 1.4, one then gets that $P\mathbf{u}$ is a λ-eigenvector. Therefore, $\mathbf{u}' = (\mathbf{u} - P\mathbf{u})/(a - b)$ is also a λ-eigenvector, and only the entries $u'_j = 1$ and $u'_l = -1$ of $\mathbf{u}'$ are not equal to 0. Using permutations $(1j)$ and (il), one gets that $\mathbf{e}_i$ for any $i \in \{1, \ldots, k\}$ is a λ-eigenvector, and since they generate U, all eigenvectors from this spectral block have the same eigenvalue.

$\boxed{3 \Longrightarrow 1}$ Suppose that we have a spectral block $\mathcal{S}$ with one degenerate eigenvalue λ. The $k - 1$ eigenvectors of this spectral block generate a subspace of eigenvectors with eigenvalue λ of dimension $k - 1$, so this subspace must be equal to U (defined as above). Notice that any permutation σ of $\{1, \ldots, k\}$ preserves U and is an identity map for eigenvectors orthogonal to U, therefore by the Proposition 1.4 it preservers the Laplacian matrix $\mathcal{L}$. □

1.2 Strong equitable partitions and synchronization

Next, we clarify the relation between symmetry orbits, equitable partitions, and the clusters identified in the first statement of Theorem 2.2. Recall that a partition of the vertex set of a graph into non-intersecting subsets $\{C_1, \ldots, C_r\}$, called *cells*, is called an *equitable partition* if for any $i, j \in \{1, \ldots, r\}$ there is a non-negative integer b_{ij} such that any vertex from C_i has exactly b_{ij} neighbors in C_j. An *external equitable partition* is defined similarly, except that one only requires the condition for $i \neq j$. For the purposes of this article, let us call a subset of vertices C an *equitable cluster* (EC) if there is an external equitable partition where C is one of the cells. We call a subset of nodes C as in Statement 1 of Theorem 1.2 a *strong equitable cluster* (SEC), that is, a subset of nodes $C \subseteq V$ such that $\mathcal{L}_{pj} = \mathcal{L}_{qj}$ for all $p, q \in C$, $j \in V \setminus C$. Equivalently, this is a subset of nodes such that every other vertex in the network is either connected to all of them (with same weights), or none at all. In turn, this is equivalent to the partition of the vertex set with cells C and $\{j\}$, $j \in V \setminus C$, being equitable. In particular, it is clear that a strong equitable cluster (SEC) is an equitable cluster (EC), but the converse is not always true.

We associate a *critical value* to each SEC, which will guarantee synchronization of the nodes in the SEC when it is reached by the coupling constant. It is defined as the sum of the second smallest Laplacian eigenvalue plus the external degree of the cluster. Formally, if C is a SEC, we define $\lambda_{\text{crit}}(C) = \lambda + d$, where λ is the second smallest eigenvalue of $L(G')$, the Laplacian of the subgraph G' induced by the nodes of C, and d is the *external degree* of C, that is, $d = \sum_{p \notin C} \mathcal{L}_{ip}$ for any $i \in C$ (this is well defined because C is a SEC).

We turn now our attention to symmetric orbits. The *orbit* of a node u is the subset of vertices $\{\sigma(u) \mid \sigma \in \mathrm{Aut}(G)\}$, that is an orbit with respect to the action the automorphism group (symmetries) of the graph G. We

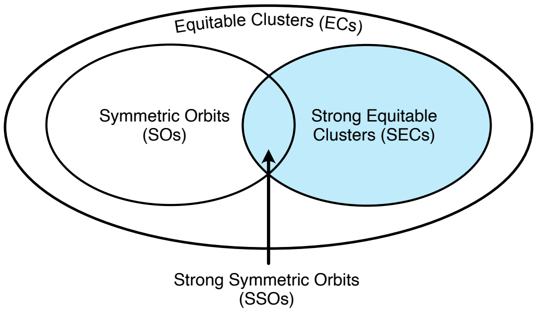

Supplementary Figure 1 – Schematic Venn diagram showing the relation between the concepts of Equitable Cluster (EC), Symmetric Orbit (SO), and Strong Equitable Cluster (SEC). Note that SOs are equitable, but not necessary strongly equitable (we call those that are, such as those in the statement of Theorem 1.3, Strongly Symmetric Orbits or SSOs). SSOs correspond to symmetric motifs [1, 4] with one orbit. Only SECs (shaded blue) support cluster synchronization in all our examples. Indeed, SECs are guarantee to achieve cluster synchronization when their critical value is reached (see main text). In addition, a SO or EC that is not a SEC on its own, but it is a SEC relative to some clusters, may support synchronization if (all vertices on) those clusters synchronize among themselves; see Supplementary Fig. 3 for an example.

call a subset of vertices C a *symmetric orbit* (SO) if it is the orbit of one (and hence all) of its vertices. It is well known that the partition of the vertices of a graph into orbits is an equitable partition. Therefore, every SO is an EC (with respect to the partition of the graph vertices into orbits), but not necessarily a SEC. Note that SOs naturally organize into symmetric motifs of support-disjoint permutations [1, 2, 4]. These are SECs if they consist of one orbit, not if they have more than one orbit (see Supplementary Fig. 3). If a SO is also a SEC, we call it a Strong Symmetric Orbit (SSO). One can show that SSOs correspond exactly to the (not necessarily basic) symmetric motifs in [1, 4] with one orbit; see Supplementary Fig. 3 for some examples. Most SOs in real-world networks are symmetric motifs with one orbit [1, 4], hence SSOs. The relation between these notions (EC, SO, SEC, and SSO) is shown diagrammatically in Supplementary Fig. 1.

In addition to SECs, which realize cluster synchronization when their critical value is reached, a EC (including a SO) which is not a SEC can also support synchronization when it has the equitable property with respect to already synchronized clusters (see Supplementary Figs. 2 and 3 for two illustrative examples). Formally, we extend the definition of SEC to a relative notion. If $C, C_1 \ldots, C_m$ are pairwise disjoint subsets of nodes, we say that C is a *SEC relative to* $C_1, \ldots, C_m$ if, for all $p, q \in C$, $\mathcal{L}_{pj} = \mathcal{L}_{qj}$ for all $j \in V \backslash (C \cup C_1 \cup \ldots \cup C_m)$ and $\sum_{j \in C_k} \mathcal{L}_{pj} = \sum_{j \in C_k} \mathcal{L}_{qj}$ for all $k = 1, \ldots, m$. An important example is an (external) equitable partition: if $C_1, \ldots, C_m$ are the cells of an external equitable partition of the graph, then each cell is a SEC relative to all the other cells. It is clear that, if each of C_1 to C_m are already synchronized ($x_i(t) = x_j(t)$ for all $i, j \in C_l$, for each $l = 1, \ldots, m$), each node in C receives an equal input from the rest of the network and thus may synchronize independently of the synchronization properties of the rest of the graph, for a high enough value of the coupling parameter.

All in all, being a SEC guarantees cluster synchronization, independently of the rest of the network, when the coupling parameter is high enough (indeed, when its critical value is reached), while being a SEC relative to clusters $C_1, \ldots, C_m$ guarantees cluster synchronization when C_1 to C_m are synchronized and the coupling parameter is high enough. (Note that, for simplicity, this discussion only considers Type II systems, see Fig. 1 of the main text.)

We can now clarify the relation between SEC and (external) equitable partition. If $C_1, \ldots, C_r$ is an equitable partition with r cells, each cell C_i is a SEC relative to the other cells. This guarantees synchronization when the critical values of all the cells are reached, but not necessarily before. In particular, it doesn't explain the order at which the cluster synchronization occurs, nor partial synchronization of, nor merger between, subsets of the equitable cells, as detected by the S_n algorithm presented in the main text.

In the theoretical and numerical analysis of the present article, all the cluster synchronization examples found correspond to SECs, or in SECs relative to synchronized clusters, confirming our intuition (see Supplementary Figs. 2, 3). We conjecture that cluster synchronization can only occur in those cases.

2 The synchronization clusters described in the main text

This final Section contains the details of the various networks considered in the main text.

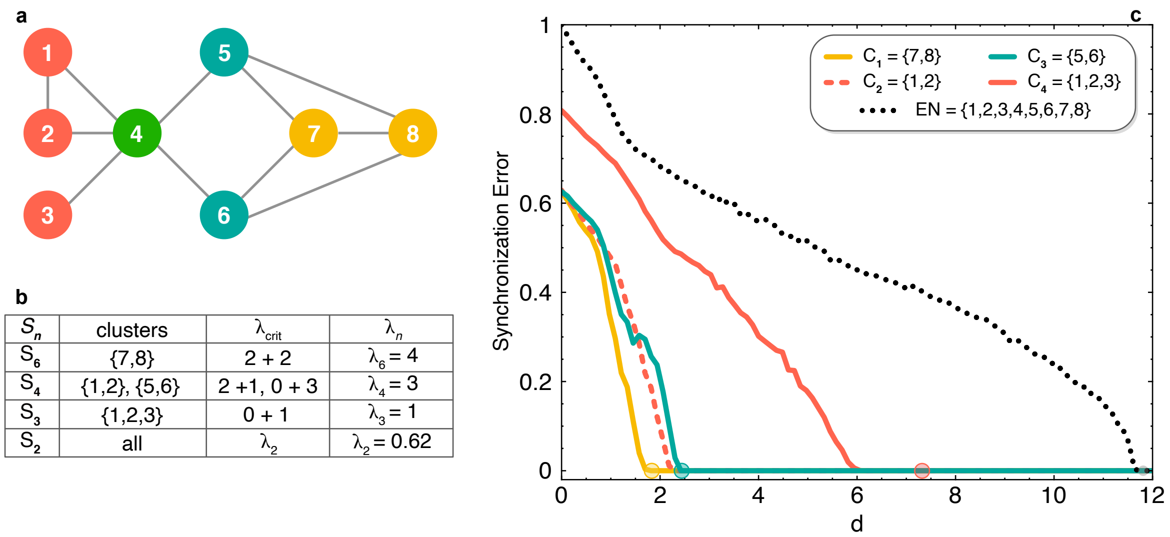

S_n	clusters	λ_{crit}	λ_n
S_6	{7,8}	2 + 2	$\lambda_6 = 4$
S_4	{1,2}, {5,6}	2 +1, 0 + 3	$\lambda_4 = 3$
S_3	{1,2,3}	0 + 1	$\lambda_3 = 1$
S_2	all	λ_2	$\lambda_2 = 0.62$

Supplementary Figure 2 – (a) A toy network with 8 nodes and 11 links, reproduced from [3]. (b) Our S_n algorithm correctly predicts cluster synchronization on clusters $\{7,8\}$, $\{1,2\}$ and $\{5,6\}$, and $\{1,2,3\}$, in that order. Note that all clusters are SECs, and all except $\{1,2,3\}$ are also SOs. For SECs, the critical eigenvalue λ_{crit} (shown as $\lambda + d$, where λ is the second smallest Laplacian eigenvalue, and d the external degree, of the induced subgraph) of each cluster, shown, determines the order at which cluster synchronization occurs (largest to smallest). The Laplacian eigenvalues of this toy network, $\lambda_1 \leq \ldots \leq \lambda_8$, are $0, 0.63, 1, 3, 3, 4, 4, 6.37$ (up to 2 decimal places) and, as predicted in Theorem 1.2, λ_{crit} coincides with λ_n at the point when the corresponding 2-block in the matrix S_n appears, as shown on the table above. Complete synchronization corresponds to the second smallest Laplacian eigenvalue of the whole network, $\lambda_2 = 0.62$ (2 decimal places). (c) Synchronization error for each of the predicted clusters (color-code in the legend). Simulations are performed with identical Lorenz chaotic oscillators, and with the same parameters and initial conditions used for generating Fig. 3a of the main text.

First, we report here below the adjacency matrix A of the network depicted in Figure 2a of the main text, which is

$$A_{ij} = \begin{bmatrix} 0 & 0.1 & 0.1 & 0.1 & 0.1 & 0.1 & 0.1 & 0.1 & 0.1 & 0.1 \\ 0.1 & 0 & 0.1 & 0.1 & 0.1 & 0.1 & 0.1 & 0.1 & 0.1 & 0.1 \\ 0.1 & 0.1 & 0 & 0.1 & 0.1 & 0.1 & 0.1 & 0.1 & 0.1 & 0.1 \\ 0.1 & 0.1 & 0.1 & 0 & 0.529 & 0.529 & 0.529 & 0.529 & 0.529 & 0.529 \\ 0.1 & 0.1 & 0.1 & 0.529 & 0 & 0.529 & 0.529 & 0.529 & 0.529 & 0.529 \\ 0.1 & 0.1 & 0.1 & 0.529 & 0.529 & 0 & 0.529 & 0.529 & 0.529 & 0.529 \\ 0.1 & 0.1 & 0.1 & 0.529 & 0.529 & 0.529 & 0 & 1.029 & 1.029 & 1.029 \\ 0.1 & 0.1 & 0.1 & 0.529 & 0.529 & 0.529 & 1.029 & 0 & 1.029 & 1.029 \\ 0.1 & 0.1 & 0.1 & 0.529 & 0.529 & 0.529 & 1.029 & 1.029 & 0 & 1.029 \\ 0.1 & 0.1 & 0.1 & 0.529 & 0.529 & 0.529 & 1.029 & 1.029 & 1.029 & 0 \end{bmatrix}$$

Second, we report the output of the application of the method described in the main text to the PowerGrid network.

In the first column of the list, we report the values of λ at which an event of cluster formation occurs along the transition, and the corresponding values of $1/\lambda$ which (once multiplied by ν^*) give the critical coupling strength's values at which such event has to be observed. Each event consists of the simultaneous emergence of synchronization clusters, and the nodes forming each one of them are reported in the second column of the list.

The 6 clusters that are highlighted in red are those whose synchronization error is reported in Fig. 5 of the main text, for a Rössler chaotic system with parameters and coupling function such that it belongs to Class II, displaying $\nu^* = 0.179$. The predicted critical coupling strengths $d_1, \ldots, d_6$ reported in the horizontal axes of Fig. 5 of the main text are, therefore:

- $d_1 = 0.179 * 0.25 = 0.04475$
- $d_2 = 0.179 * 0.333 = 0.0596$

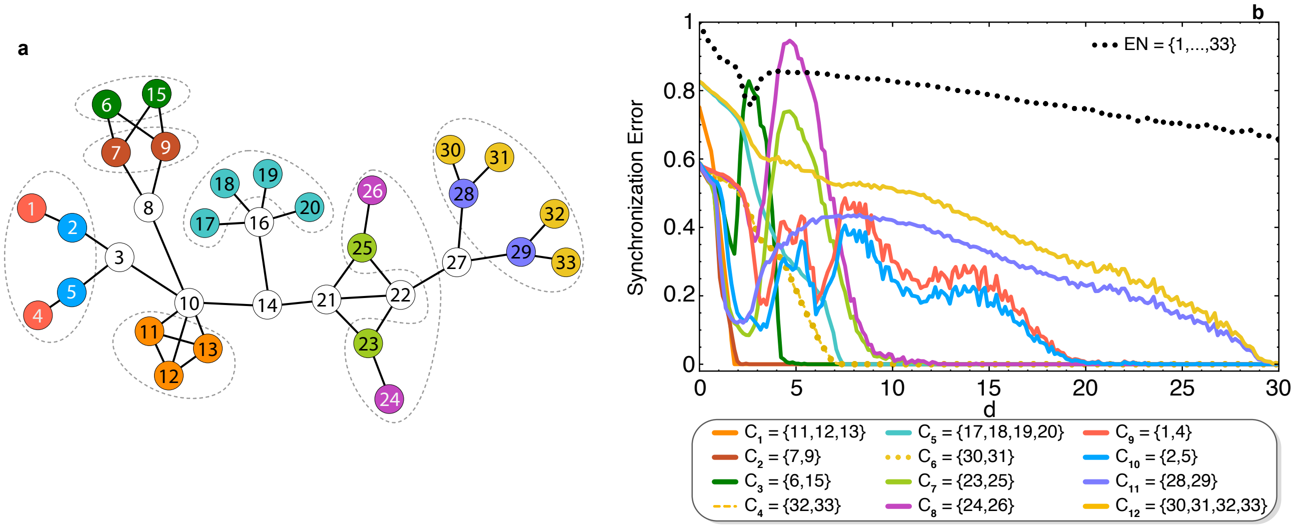

Supplementary Figure 3 – (a) A toy network with 33 nodes and 39 links, reproduced from [4], showing small examples of typical real-world symmetric motifs (subgraphs where the network symmetry is locally generated [1, 4]), shown inside the dotted lines. Each symmetric motif is composed of one or more vertex orbits (SOs in our terminology), shown by color. Of the 10 SOs in this toy example, 4 are SECs (dark green, brown, orange, and turquoise), corresponding to symmetric motifs with one orbit, and 6 are relative SECs: the red orbit is a SEC relative to the dark blue orbit (and vice-versa), the purple orbit relative to the green orbit (and vice-versa), and the yellow orbit relative to the pale blue orbit (and vice-versa). Notably, the yellow orbit contains two SECs, namely the clusters $\{30, 31\}$ and $\{32, 33\}$, separately. The Laplacian eigenvalues of this toy network, $\lambda_1 \leq \ldots \leq \lambda_{33}$, are 0.0, 0.04, 0.11, 0.14, 0.23, 0.27, 0.38, 0.42, 0.59, 1.0, 1.0, 1.0, 1.0, 1.0, 1.37, 1.7, 2.0, 2.09, 2.49, 2.62, 2.72, 3.0, 3.41, 3.73, 3.97, 4.0, 4.0, 4.42, 5.09, 5.25, 5.53, 6.11, and 7.3 (up to 2 decimal places). (b) Our S_n algorithm correctly predicts cluster synchronization on the clusters shown, in the order shown. Note that the four SOs that are SECs become synchronized at their critical value λ_{crit} (shown as $\lambda + d$, as in Supplementary Fig. 2), plus $\{30, 31\}$ and $\{32, 33\}$, which are SECs but not SOs. As expected, the critical value λ_{crit} of each SEC corresponds to the nth smallest Laplacian eigenvalue of the whole network, λ_n, with $\lambda_2 = 0.04$ (2 decimal places) achieving global synchronization. The three relative SECs also become synchronized after, or at the same time as (depending on the relative values of the critical values) the clusters they are SECs relative to. Indeed, the red cluster becomes synchronized at the same time as the dark blue cluster, and the same for the purple and green clusters. For the yellow orbit, note it contains two (structurally identical) SECs ($\{30, 31\}$, and $\{32, 33\}$), which synchronize separately when their critical eigenvalue is reached, but their union, which is not a SEC, rather a SEC with relative to the light blue orbit, only synchronizes when the light blue orbit does. The predicted sequence of events is fully confirmed in our simulations.

- $d_3 = 0.179 * 0.5 = 0.0895$
- $d_4 = 0.179 * 0.723 = 0.1294$
- $d_5 = 0.179 * 1 = 0.179$
- $d_6 = 0.179 * 1.707 = 0.3056$

As already discussed in the main text, a total of 381 clusters are found, involving an overall number of 871 network's nodes which get clustered during the transition: 310 clusters contain only 2 nodes, 49 clusters are made of 3 nodes, 14 clusters are formed by 4 nodes, 4 clusters have 5 nodes, 2 clusters appear with 6 nodes, 1 cluster has 7 nodes, and 1 cluster is made of 9 nodes.

The list reported here below is limited to the first 11 predicted events.

λ	Clusters
$\lambda = 4$<br>$\frac{1}{\lambda} = 0.25$	[1081,1082] [2638,2640,2641] [2793,2794] [3037,3038] [3089,3090,3092] [3220,3222] [3226,3227] [3249,3250] [3252,3254] [3297,3298,3300] [3349,3350,3351]
$\lambda = 3$<br>$\frac{1}{\lambda} = 0.333$	[346,347] [2012,2013] [2153,2154] [2442,2443] [2452,2453] [2689,2690] [2917,2918] [3057,3058] [3065,3069] [3067,3068] [3075,3076] [3198,3199] [3260,3262] [3283,3284] [3299,3301] [3312,3313] [3319,3320] [3325,3326] [3359,3360] [3651,3652] [4480,4481]
$\lambda = 2$<br>$\frac{1}{\lambda} = 0.5$	[7,8] [122,123] [341,342] [632,633] [638,639] [641,643] [668,781] [860,861] [966,967] [1154,1155] [1476,1478] [1829,1834] [1956,1960] [1957,1961] [1968,2125] [2111,2112] [2184,2186] [2196,2262] [2221,2222] [2284,2285] [2318,2320] [2469,2470] [2489,2490] [2523,2830] [2655,2656] [2664,2665] [2813,2814] [2829,2834] [2841,2842] [2881,2882] [2929,2935] [3030,3031] [3185,3187,3188][3419,3420] [3474,3475] [3538,3539] [3554,3555] [3557,3558] [3726,3727] [3804,3805] [3902,3903] [4162,4163] [4455,4457] [4868,4923]
$\lambda = 1.753$<br>$\frac{1}{\lambda} = 0.57$	[582,586] [585,587] [635,636]
$\lambda = 1.382$<br>$\frac{1}{\lambda} = 0.723$	[1344,1345] [1825,1826][1835,1836][2256,2257]

$\lambda = 1$<br>$\frac{1}{\lambda} = 1$	[12,4935] [14,173] [19,21] [32,34] [36,37] [66,154] [86,87,88] [101,102] [109,4510] [133,135,136] [206,207] [213,214,215,216] [218,4523] [248,249] [256,257,258] [284,285] [348,391] [355,357,360,361] [405,406] [416,417] [419,420] [435,436] [461,462,463,464] [468,469] [573,575] [591,592] [593,646] [596,597] [601,4519,4520,4521,4522] [605,607] [647,648] [654,655,656] [683,689] [690,703,704,705,706,707,708] [693,694] [699,700,702] [709,712] [717,719] [759,771] [778,779] [783,784,785] [806,807] [808,809] [825,836] [839,885] [841,842] [858,859] [870,878] [872,874] [912,913] [934,935,939,940,941,942,943,944,945] [981,982,983,984,985] [987,988,989] [1019,1020] [1021,1022] [1025,1026] [1027,1028] [1124,1126] [1131,1132,1133] [1142,1144,1145,1146] [1209,1565,1570] [1228,1230] [1394,1395] [1419,1420] [1481,2276] [1535,1536] [1767,1768] [1837,1838] [1840,1841] [1917,1921] [1935,1936] [2034,2139] [2060,2062] [2169,2170] [2249,2250] [2268,2269] [2272,2273] [2286,2442,2443] [2352,2354] [2386,2387] [2401,2402,2403] [2414,2415] [2449,2450] [2462,2463,2464] [2497,2498] [2553,2555,2562,2563] [2569,2571] [2576,2578] [2635,2636] [2642,2643] [2671,2672] [2705,2706] [2709,2710,2711] [2725,2726,2728] [2730,2731] [2742,2743] [2744,2746] [2748,2749] [2758,2759] [2835,2836,2838] [2839,2840] [2843,2844] [2872,2873] [2904,2905] [2910,2913,2914,2915] [2979,2980] [2997,2998] [3000,3001] [3004,3005,3006,3007,3008,3009] [3011,3012] [3023,3024] [3041,3042,3189,3190] [3054,3268] [3065,3067,3068,3069] [3075,3076,3077] [3080,3081,3082,3083] [3084,3311] [3088,3089,3090,3092] [3104,3105] [3196,3218,3219] [3201,3202] [3260,3261,3262] [3283,3284,3285] [3286,3287] [3288,3289] [3297,3298,3299,3300,3301] [3309,3310] [3318,3347,3364] [3327,3328] [3349,3350,3351,3369] [3363,3365] [3398,3399] [3410,3412,3413,3724] [3435,3436,3437] [3464,3465] [3480,3481] [3484,3485] [3559,3561] [3572,3577,3578] [3609,3632,4918] [3611,3613,3614] [3616,3730] [3619,3620] [3622,3629] [3624,3704] [3625,3801] [3630,3705,3706] [3639,3640] [3658,3660] [3661,3662,3666] [3700,3812] [3717,3718] [3778,3796] [3802,3818] [3809,3810] [3814,3838] [3840,3841] [3878,3879] [3882,3885] [3938,3944] [3946,3947,3948] [4097,4098] [4107,4174] [4110,4111,4113] [4125,4126] [4155,4156] [4171,4172,4173] [4175,4181] [4177,4179] [4195,4197] [4211,4213] [4217,4218,4219] [4223,4224,4225] [4232,4233] [4252,4253] [4262,4263] [4264,4266] [4270,4271] [4282,4283] [4287,4288,4289] [4303,4304,4305] [4307,4308,4309,4310] [4311,4312,4313] [4320,4321] [4325,4326] [4328,4329] [4330,4331] [4336,4337,4338,4339] [4340,4341] [4346,4347,4348,4349] [4351,4352,4353]
	[4354,4355] [4357,4358,4359,4360,4361] [4362,4363] [4365,4366,4367,4368,4369] [4370,4371,4372,4373] [4377,4378,4379,4380,4381,4382] [4384,4385] [4387,4388] [4389,4390,4391] [4393,4394] [4395,4396,4397] [4398,4399] [4400,4401] [4488,4489] [4511,4512] [4513,4515] [4516,4518] [4604,4605] [4643,4647,4648,4649] [4658,4659] [4679,4680,4681] [4684,4685] [4702,4703] [4717,4718,4719] [4729,4730] [4746,4860] [4799,4813] [4800,4802] [4829,4830] [4841,4842] [4843,4844] [4884,4885] [4907,4908]

$\lambda = 0.6972$ $\frac{1}{\lambda} = 1.434$	[2774,2939] [2775,2940] [4826,4828] [4827,4850]
$\lambda = 0.6228$ $\frac{1}{\lambda} = 1.606$	[614,615] [617,618] [622,623]
$\lambda = 0.5858$ $\frac{1}{\lambda} = 1.707$	[564,565,566] [2510,2949] [2511,2627] [2798,2799] [2831,2832,2833] [2852,2853,2854] [2911,2912] [2946,2947] [2964,2965] [3128,3177] [3208,3209] [3211,3212,3213] [3233,3234,3235] [3244,3245,3246] [3279,3280] [3372,3373]
$\lambda = 0.5188$ $\frac{1}{\lambda} = 1.927$	[3742,3746,3749][3743,3748,3750][3744,3745,3747]
$\lambda = 0.3820$ $\frac{1}{\lambda} = 2.618$	[868,907] [869,904] [1568,1571] [1616,2045] [2750,2774,2939] [2751,2775,2940] [2891,3043] [2892,2894] [2897,2901] [2898,2900] [3146,3150] [3147,3151] [3148,3272] [3149,3273]

Third, we report details of the other two real-world networks which were analyzed in Figure 6 of the main text.

The Yeast protein-protein interaction network [5] is a dataset made of $N = 1,647$ nodes and $E = 2,518$ edges. During the transition to synchronization, 188 clusters are found, and Figure 6 of the main text refers to 4 of them: C_1 and C_2 (which are both 2 nodes clusters), C_3 (a cluster containing 3 nodes), C_4 (a cluster of 7 nodes). On the other hand, the ego-Facebook network [6] is a dataset containing $N = 2,888$ nodes and $E = 2,981$ edges. 18 clusters are found during the transition to synchronization, and once again we focused on 4 of them: C_1 and C_2 (which are both made of 2 nodes), C_3 (a cluster containing 5 nodes), C_4 (a subset of 3 nodes - out of a cluster of 280 nodes - which is forming a cluster by itself). The predicted values of the critical coupling strengths are reported, as colored points, in the horizontal axis of Figures 6a and 6b of the main text.

Supplementary References

[1] B.D. MacArthur, R.J. Sánchez-García, and J.W. Anderson, Symmetry in complex networks. Discrete Applied Mathematics 156 (18), 3525-3531 (2008).

[2] B.D. MacArthur, and R.J. Sánchez-García, Spectral characteristics of network redundancy. Physical Review E, 80, 026117 (2009).

[3] O'Clery, N., Yuan, Y., Stan, G.B., and Barahona, M. Observability and coarse graining of consensus dynamics through the external equitable partition. Physical Review E, 88(4), 042805 (2013).

[4] R.J. Sánchez-García, Exploiting symmetry in network analysis. Nature Communications Physics 3, 87 (2020).

[5] H. Yu, P. Braun, M.A. Yildirim, I. Lemmens et al., High-Quality Binary Protein Interaction Map of the Yeast Interactome Network, Science 322, 104-110, (2008).

[6] J. McAuley and J. Leskovec, Learning to discover social circles in ego networks, Proceedings in Advances in Neural Information Processing Systems, 548–556 (2012).
